# Acute Urinary Retention in the Male Child from Urethral Calculi: A Report of Three Cases

**DOI:** 10.1155/2019/5762139

**Published:** 2019-05-14

**Authors:** L. O. Mbouché, G. Ondobo Andzé, A. S. Nwaha Makon, D. Nyanit Bob, E. Njuma Tamufor, A. L. Amenglé, F. F. Mouafo Tambo, P. Y. Mure, J. Zé Mikandé, F. F. Angwafo

**Affiliations:** ^1^Yaounde Gyneco-Obstetric and Pediatric Hospital, Cameroon; ^2^Faculty of Medicine and Biomedical Sciences of University of Yaoundé I, Cameroon; ^3^University Teaching Hospital of Lyon, France

## Abstract

Urinary stones are uncommon in children. Urethral location of calculi can give rise to various clinical manifestations. We report three cases of urethral lithiasis presenting with acute urinary retention in children.

## 1. Introduction

Acute urinary retention is commonly encountered in urological practice in adults, mostly from prostatic pathologies. In the male child, lower urinary tract symptoms are usually secondary to congenital uropathy, most often posterior urethral valves, and sometimes neurogenic bladders from spinal dysraphism. Urinary stones can be an obstacle to the normal flow of urine at any level of the lower urinary tract. In Europe, the prevalence of urolithiasis varies from 5 to 9% [1]. It is 20 times rarer in children than in adults [2]. Urolithiasis prevalence varies from 7 to 13% in North America; in Asia its prevalence varies from 1 to 5% [1]. There is a pediatric stone belt that runs from the Philippines, Myanmar, and Thailand in the Far East, extending to Pakistan and Iran in the Middle East and up to Turkey [3].

In sub-Saharan Africa, the prevalence of lithiasis in children is variously appreciated according to the geographical location although literature is sparse and limited to hospital studies. In Cameroon, a series of 123 cases in two hospitals reported a prevalence of 17% in children aged from 7 to 15 years, including 2% of stones located in the urethra [4]. However, no cases of acute retention of urine were reported.

The article presents three cases of acute urine retention from stones located in the male urethra.

## 2. Presentation

### 2.1. Case 1

A 2-year-old male was referred to the Pediatric Surgery Department of the Yaoundé Gynaeco-Obstetric and Pediatric Hospital from the Western region of Cameroon, with an enlarged bladder, dribbling urine with overflow incontinence. He presented with progressive dysuria from age 3 months, without fever or hematuria, but with repeated urinary tract infections.* Proteus vulgaris* sensitive to ceftriaxone was isolated in one of the urine samples. He was circumcised at 6 months and there was no family history of urolithiasis.

**Figure 1:**
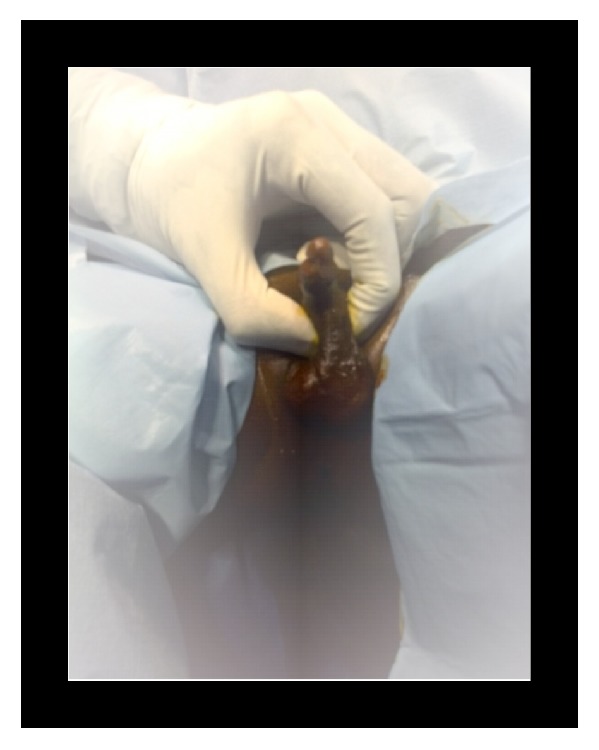
Palpation of mass at the base of the penis (case 1).

**Figure 2:**
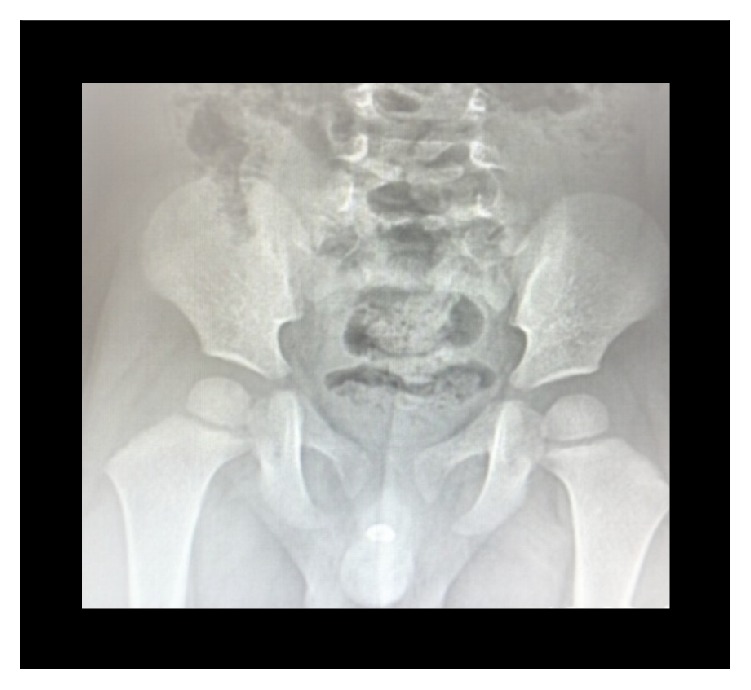
Plain abdominal radiograph showing the urethral calculus (case 1).

**Figure 3:**
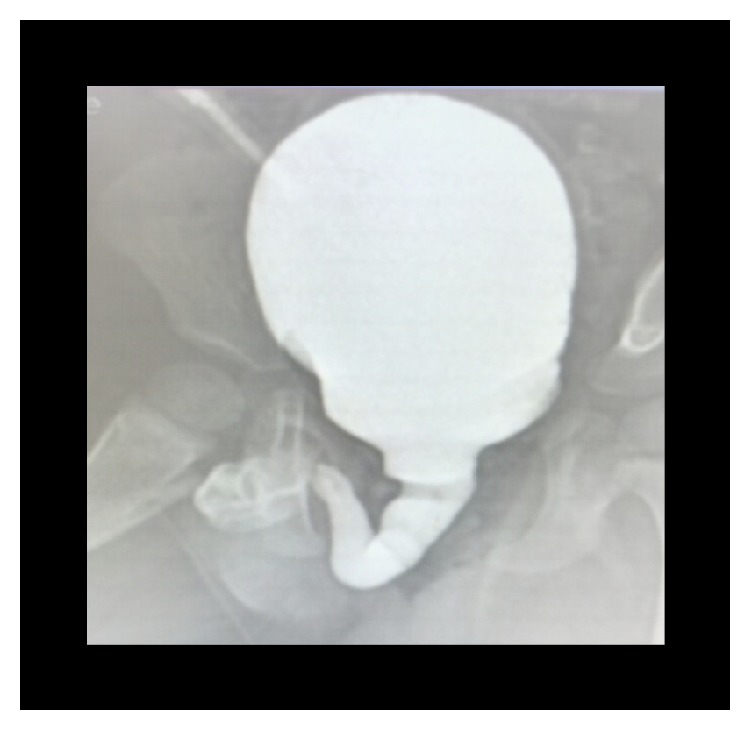
VCUG (elongated, dilated urethra) (case 1).

**Figure 4:**
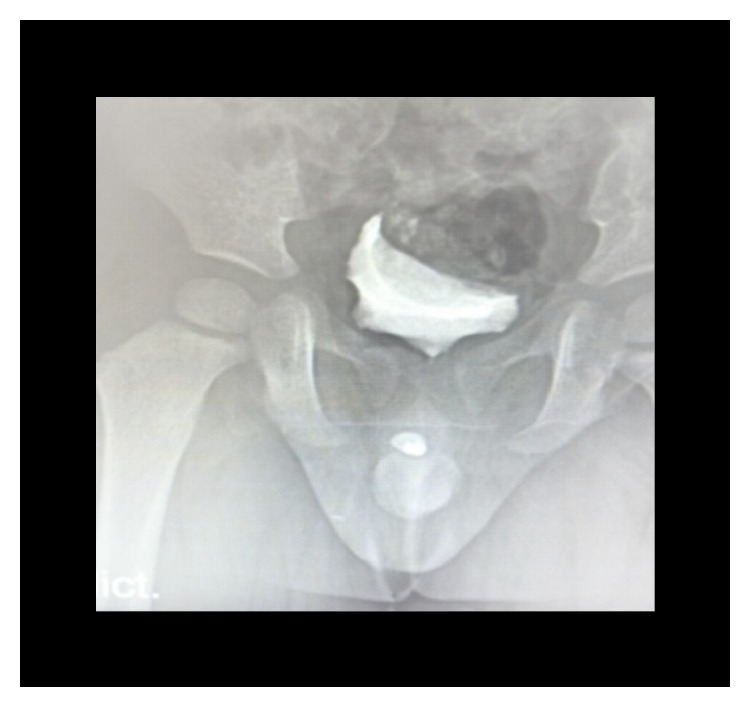
Postvoid residue on VCUG (case 1).

On clinical examination, the infant was a well-developed, well-nourished male, with a temperature of 37.5°C, a heart rate of 106 beats per minute, a respiratory rate of 25 cycles per minute, and a weight of 12 kg. A tense and sensitive bladder was palpated in the suprapubic region as well as a hard mass, at the base of the penis (Figure 1). There was no costovertebral angle tenderness. An abdominal scout film showed a rounded opacification beneath the pubic symphysis (Figure 2). Voiding radiographs confirmed interruption to flow in the penile urethra with proximal urethral dilatation (Figure 3). The bladder was markedly distended; there was no vesicoureteral reflux. The postvoid residual volume was significant (Figure 4). Ultrasonography of the urinary tract did not reveal abnormalities in the upper unit. Urine culture revealed* E. coli *sensitive to ceftriaxone. Blood count, urea, and creatinine were normal.

**Figure 5:**
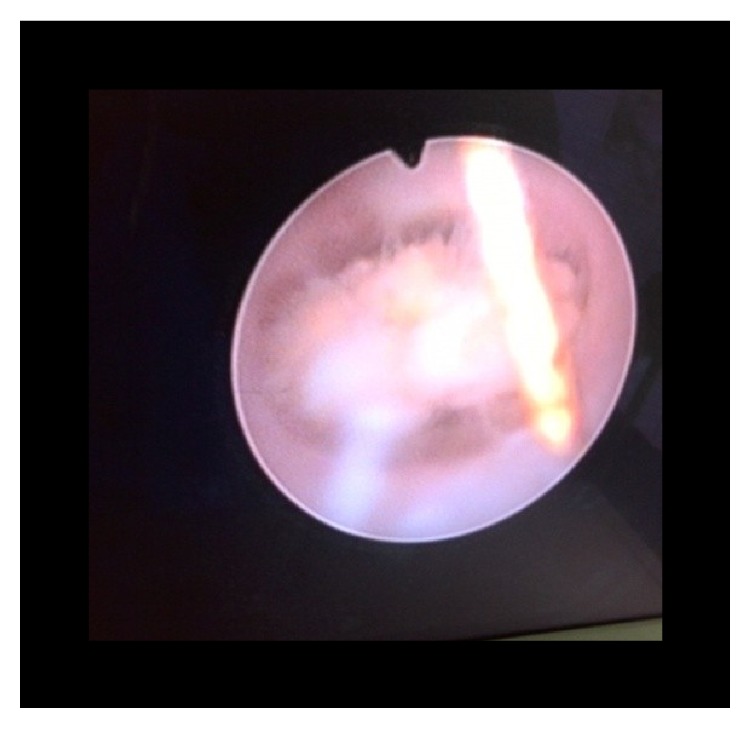
Endoscopic view of the calculus (case 1).

**Figure 6:**
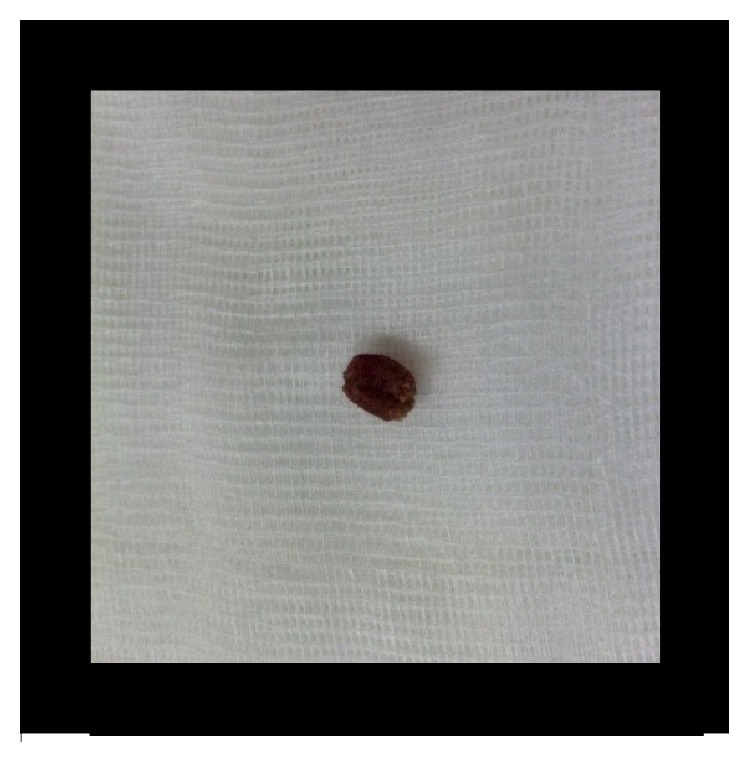
Extracted urethral stone (case 1).

A suprapubic cystostomy was performed at bedside. At urethroscopy, a stone completely obstructing the lumen and encrusted in the urethral wall, prevented endoscopic extraction (Figure 5). Urethrotomy was therefore performed. An elongated 6 mm x 9 mm lithiasis with a crystalline surface was extracted (Figure 6). A Foley catheter (Ch 10) was left in place for 21 days.

The postoperative period was normal and the infant was discharged from hospital with normal voiding. He has remained asymptomatic at 1-, 3-, and 6-month follow-up visits.

The results of infrared spectrophotometric analysis of the calculus are as follows:

- Nucleus: urate ammonium acid (15%), calcium oxalate monohydrate (70%), and calcium oxalate dihydrate (15%)
- Surface: calcium oxalate dihydrate (100%)

A urinary metabolic assessment for hyperoxaluria will complete the work-up as soon as funding is available.

### 2.2. Case 2

**Figure 7:**
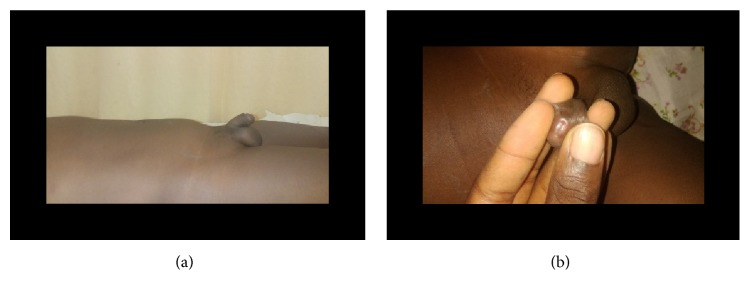
(a) Suprapubic bladder distension (case 2); (b) meatal stenosis (case 2).

**Figure 8:**
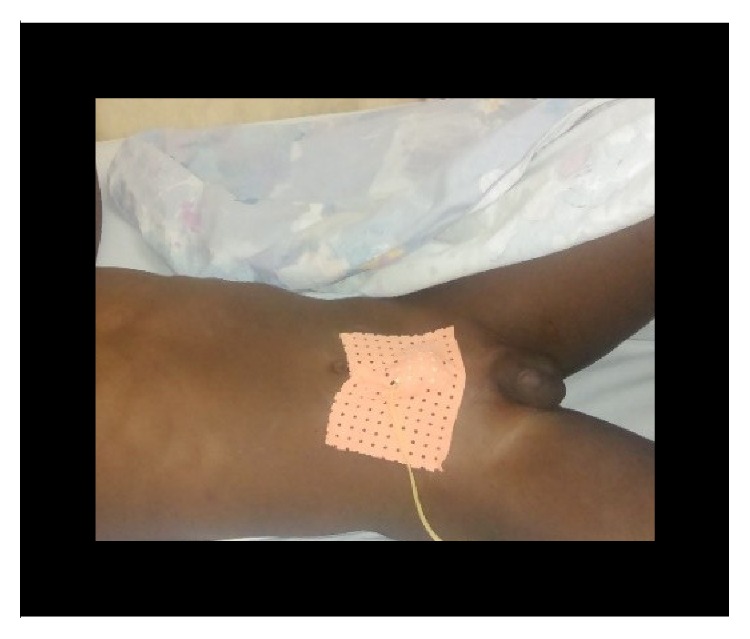
Suprapubic cystostomy in place (case 2).

**Figure 9:**
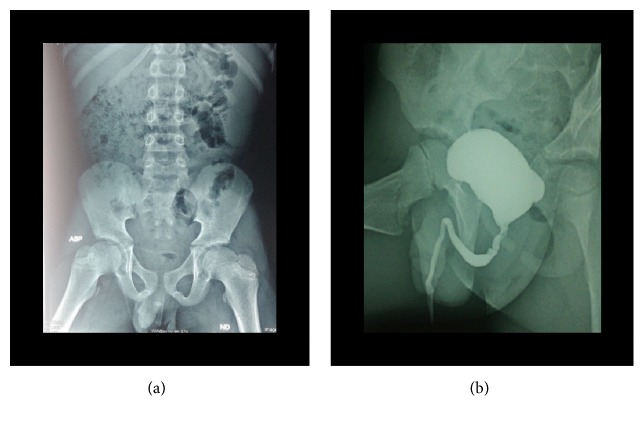
(a) Scout film, stone in the penile midshaft (case 2). (b) VCUG, partial urethral obstruction (case 2).

**Figure 10:**
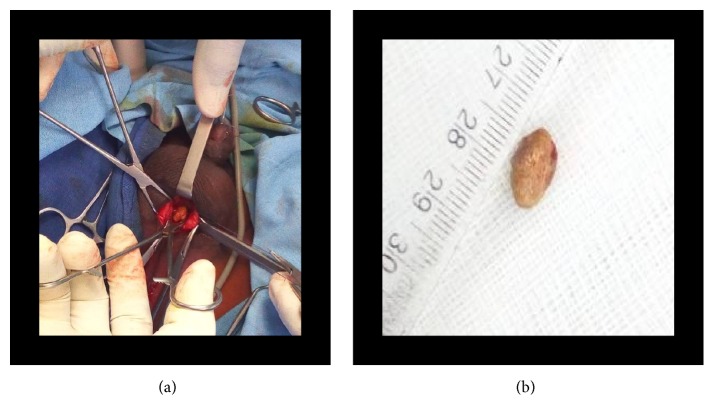
(a) Urethral calculus, in situ (case 2). (b) Extracted calculus (case 2).

A fourteen-year-old boy presented at the Emergency Department of the Yaoundé Gynaeco-Obstetric and Pediatric Hospital complaining of inability to urinate for over 20 hours despite the sensation of a full bladder. He was born at term, had normal urine flow at birth, and was treated for a urinary tract infection at 9 months. He was circumcised at 9 years of age without complications. The present symptoms began 1 month previously with pollakiuria and painful micturition with no fever or hematuria. Physical examination revealed an agitated child, writhing with pain in the hypogastric region, and a palpable full bladder (Figure 7). Vital signs were normal. There was no costovertebral angle tenderness. The testes were both descended and the penis presented a stenotic meatus. Palpation of the body of the penis did not reveal any masses. 600 ml of clear urine was drained from an urgent bedside 8 Fr cystostomy (Figure 8). However urinalysis was positive for nitrites and leukocyturia. Blood urea nitrogen and creatinine were normal; there was marked leukocytosis at 11000/mm^3^. An abdominal ultrasound showed grade 2 right ureterohydronephrosis. A scout film confirmed an ovoid calculus in the proximal part of the anterior urethra (Figure 9). A voiding cystourethrogram showed a partially obstructed urethra and absence of passive as well as active vesicoureteral reflux (Figure 10). The patient received amoxicillin-clavulanic acid. Definitive management under general anaesthesia consisted of meatotomy and open perineal urethrostomy. An encrusted urethral stone (13 mm x 6 mm) with a speculated surface was extracted (Figure 10). An indwelling Foley catheter (12 Fr) was left in place for 10 days. The patient was given Oxybutynin for bladder spasms and amoxicillin-clavulanic acid as long as the indwelling catheter was in place.

### 2.3. Case 3

**Figure 11:**
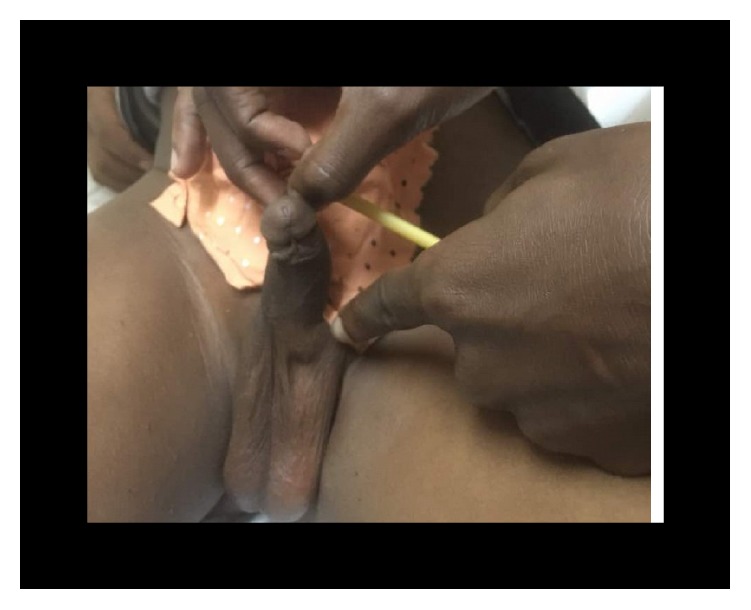
Physical exam showing location of stone (case 3).

**Figure 12:**
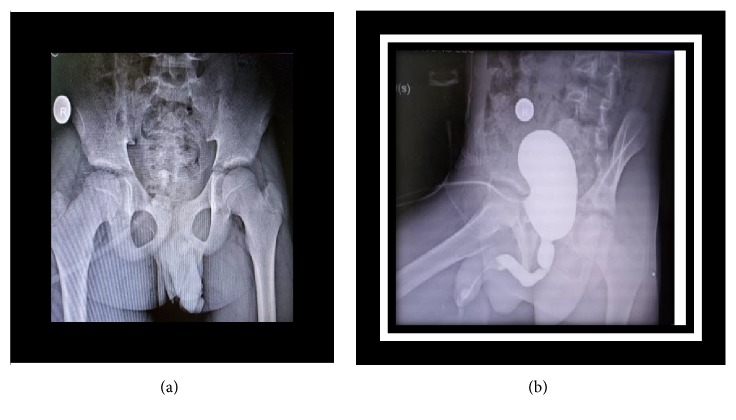
(a) Scout film showing penile stone (case 3). (b) VCUG, interruption of contrast (case 3).

**Figure 13:**
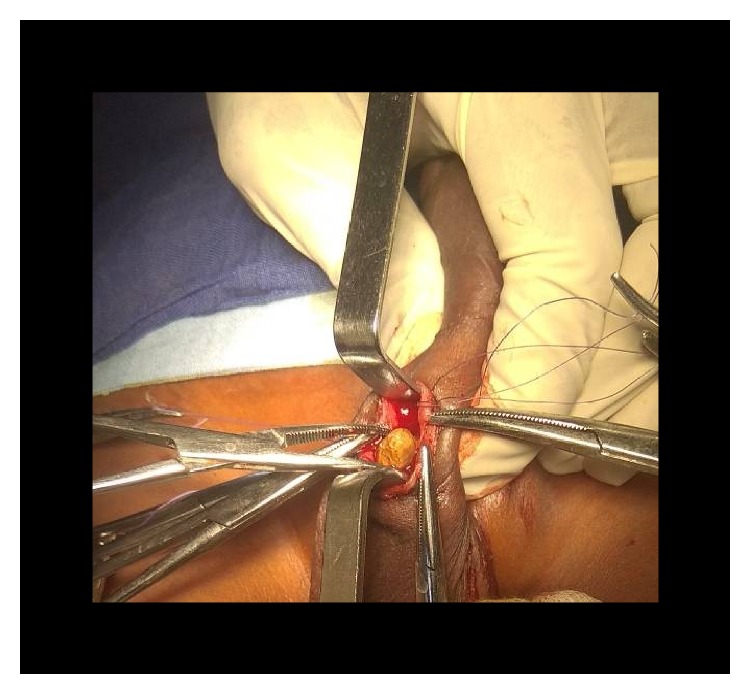
Urethral stone extraction (case 3).

**Figure 14:**
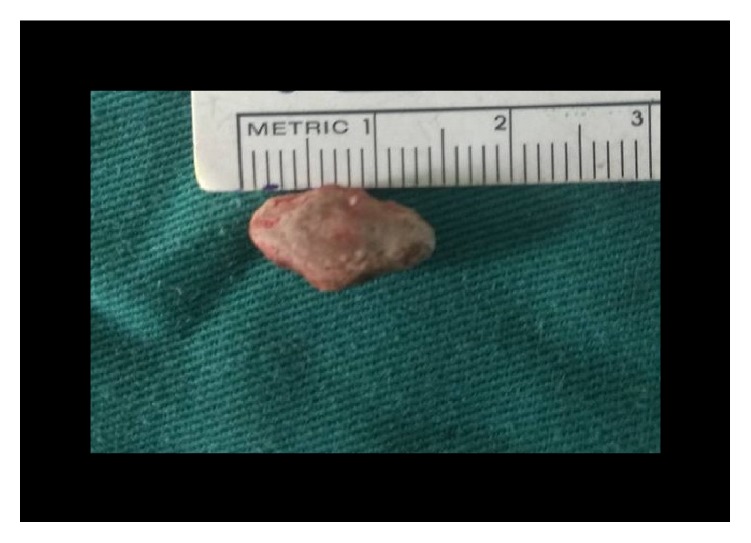
Stone removed (case 3).

**Figure 15:**
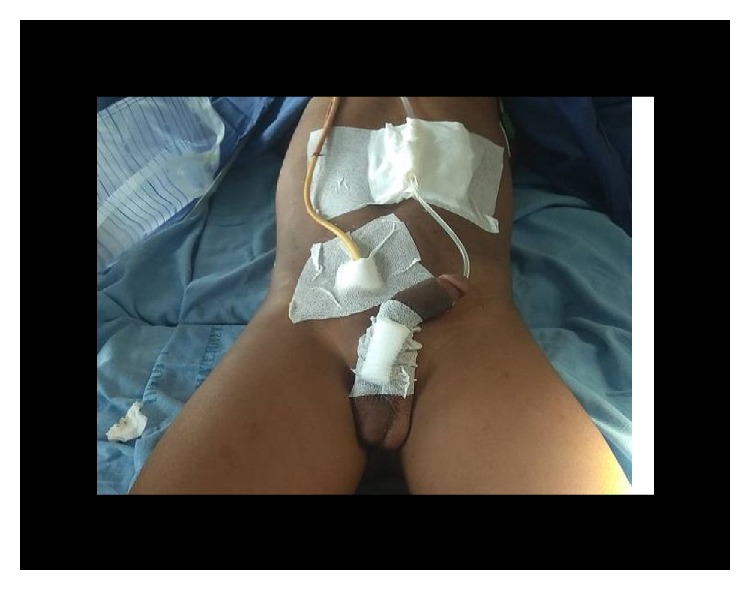
Postoperative dressing with urethral and suprapubic catheter (case 3).

A patient of 11 years old was referred to the service for acute urinary retention, following repetitive failure to insert urethral catheter two weeks after trauma to the genitalia during a football match. He complained of difficult and painful micturition and eventually urinary retention. A cystostomy was placed in the emergency room. He was circumcised at age 3 and was treated for recurrent UTI in the past. On physical examination, the urethra meatus was normal; there was a hard, round, palpable mass in a tender penile urethra (Figure 11). A scout film of the pelvis showed an image of a urethral stone (Figure 12(a)). Opacification of lower tract during VCUG confirmed the obstruction from the stone but no reflux (Figure 12(b)). There was marked leukocyturia at 17000/mm3.* Citrobacter koseri* was isolated from the urine. We removed the calculus via urethrotomy (Figures 13 and 14). The two urinary catheters were kept on the abdomen after dressing (Figure 15). Postoperative follow-up was normal.

## 3. Discussion

Urolithiasis is rare in children [2]. It accounts for 5 to 15% of urinary stones in developing countries [5, 6]. The series from two geographic zones from Cameroon showed that urate stones are not uncommon as reported in other developing countries. The said series represented a tip of the iceberg as issues of geographical, cultural, and financial access to care limited case recruitment. It generally affects young boys under 5 years of age and bladder calculi are most frequent. Most stones that are impacted in the urethra are indeed of bladder origin. Urethral stones account for 0.3% of all urolithiasis [7]. These stones are constituted mainly of urates and/or triple (Ammonium, Magnesium, and Calcium) phosphates [8, 9].

Pain and a mass in the penis is a common clinical presentation. The signs depend on the location and the size of the stone. Calculi of the posterior urethra are often larger and urine can flow around them. Smaller stones tend to localize at the distal part, most frequently in the fossa navicularis often completely obstructing the passage causing acute retention of urine [10].

Posterior urethral valves are the most common cause of urinary retention in the male child. The cases presented underline the necessity of performing a complete physical examination of the genitalia, including the inspection, percussion, and palpation of the penis in search of a calculus. If urethral lithiasis is suspected, a plain X-ray of the abdomen may be useful to confirm the diagnosis and location of the stone. The sensitivity of pediatric ultrasound for the detection of stones varies from 59 to 78% with a specificity approaching 100% [11–13]. Its use at the level of the urethral is not popular but would certainly be advantageous over standard X-ray which is increasingly proscribed in children.

From a biochemical standpoint, ammonium urate is responsible for nearly half of the calculi observed in developing countries. It constitutes approximately 50% of the nidus of these calculi. In developing countries, a large proportion of urate-nucleated calculi is caused by an infectious process and potentiated by several mechanisms: a gastrointestinal infection that causes diarrheal episodes with loss of electrolytes such as sodium and potassium; an infection of the urinary tract from urease-producing bacteria; or even a mixed infection (both digestive and urinary) [8]. Also, it has been documented that the incidence of urinary stones is higher in countries with warm or hot climates, probably due to low urinary output and scanty fluid intake. These are some of the factors that contribute to the geographical pattern that characterizes the North American and Afro-Asian stone belts [14]. The Afro-Asian stone belt stretches from Sudan, the Arab Republic of Egypt, Saudi Arabia, the United Arab Emirates, the Islamic Republic of Iran, Pakistan, India, Myanmar, Thailand, and Indonesia to Philippines. Within a stone belt, the incidence of urolithiasis varies within the regions due to the local physical geographic conditions and dietary/behavioural practices [15]. The reported prevalence of uncommon stone types could be an underestimation in many developing countries due to the unavailability of qualified manpower and diagnostic tools [15].

Endoscopic lithotripsy has become the standard treatment in the management of bladder stones and urethral lithiasis. The latter is achieved after pushing back the stone into the bladder with success rates close to 80% [7]. In all three patients, in the absence of lithotripsy and in the presence of large obstructing calculi encrusted in the urethra, open ureterolithotomy was indicated. They all void normally with follow-up that varies from 6 months to 4 years. Lifelong follow-up in respect of recurrent urinary infections and stones but most especially urethral stricture is imperative. North-south collaboration is often necessary for the evaluation of the stone chemical composition in resource limited areas.

## 4. Conclusion

An underlying congenital or acquired pathology should be sought in the male child presenting with acute urinary retention. The urethra should be examined for an obstructing stone. The association of a plain abdominal X-ray and an ultrasound not only permits the identification of calculi but also appreciates the impact of obstruction on the upper urinary tract. Urgent urinary diversion and removal of the calculus with minimal urethral trauma is recommended treatment.
